# Agreement between spirometric quality reviewers in an epidemiological study

**DOI:** 10.36416/1806-3756/e20250173

**Published:** 2026-03-05

**Authors:** Santiago C. Arce, Ricardo Del Olmo, Adrián Ceccato, Juan M. Luchelli, Evelyn Sureda, Mariano Fielli, Sergio J. Arias, Andrés L. Echazarreta, EPOC.AR Study Group

**Affiliations:** 1. Instituto de Investigaciones Médicas Alfredo Lanari, Universidad de Buenos Aires, Ciudad Autónoma de Buenos Aires, Argentina.; 2. Hospital María Ferrer, Ciudad Autónoma de Buenos Aires, Argentina.; 3. Hospital Nacional Profesor Alejandro Posadas, El Palomar, Provincia de Buenos Aires, Argentina.; 4. Instituto Nacional de Enfermedades Respiratorias Emilio Coni, Ministerio de Salud de la Nación, Santa Fe, Argentina.; 5. Centro Médico Capital, La Plata, Provincia de Buenos Aires, Argentina.

**Keywords:** spirometry, spirometry standardization, quality control, spirometry training

## Abstract

**Objective:**

. Spirometry is the gold standard among pulmonary function tests, with acceptability criteria established to ensure reliable results. However, variability in the interpretation of spirometry by trained reviewers remains unexplored. This study assessed agreement among trained reviewers regarding spirometric acceptability and its components, including the number of acceptable maneuvers, predominant defect, and repeatability.

**Methods:**

. Spirometries from 3,982 adults (≥40 years) participating in an epidemiological study across six cities in Argentina were independently reviewed by junior reviewers (JRs) and adjudicated by senior reviewers (SRs), following the 2005 ATS/ERS guidelines. Agreement was analyzed using Cohen’s kappa, McNemar’s test, and proportion comparisons.

**Results:**

. A total of 7,964 pre- and post-bronchodilator spirometries were analyzed. Agreement on overall acceptability between JRs was 76% and 84% in two independent reviewer teams (RvTa and RvTb, respectively). Agreement on specific defects was highest for numerical criteria, such as insufficient expiratory time (k=0.61) and excessive back-extrapolated volume (BEV; k=0.52), but lower for subjective criteria, including weak effort (k=0.19) and cough in the first second (k=0.36). No agreement was observed for glottic closure (k=0). SR adjudication increased the proportion of acceptable tests from 54% to 65%.

**Conclusions:**

Reviewer variability significantly affects spirometric acceptability, with numerical criteria achieving higher agreement than subjective assessments. These discrepancies have implications for large-scale studies, data quality, and automated algorithm development. Cross-checking reviews is recommended to enhance reliability. Further standardization of acceptability criteria is warranted.

## INTRODUCTION

Spirometry is the most widely used and standardized pulmonary function test to date.^1^ ^-^ ^4^ Technological advances have simplified its execution and improved its accuracy and precision.^5^ ^-^ ^8^ Furthermore, standardized guidelines have unified the execution, review, and interpretation of spirometric tests. Nevertheless, to our knowledge, no study has specifically evaluated inter-reviewer agreement in spirometry assessment.

In clinical medicine, diagnostic reports can be based on quantitative data, qualitative data, or both.^9^ Spirometry reports rely on numerical measurements (quantitative), but the evaluation of acceptability and maneuver selection-factors that determine the final results-includes both quantitative and qualitative components. Some acceptability criteria depend on numerical thresholds (e.g., expiratory time, back-extrapolated volume), whereas others require visual inspection and reviewer validation (e.g., curve free from artifacts, cough affecting results). Although spirometric acceptability criteria are well established, subjectivity among reviewers may influence the final outcome.

The objective of the present study was to assess inter-reviewer agreement regarding overall spirometric acceptability and its individual components. 

## METHODS

As part of an epidemiological study conducted in 2016,^10^ spirometry was performed on 3,999 volunteers from six cities in Argentina, across three successive phases involving two cities at a time (see Tables 1 and S1 for population characteristics and Table S2 for cities and referral centers). Tests were carried out using spirometers equipped with an ultrasonic sensor (EasyOne, NDD; Zurich, Switzerland), according to standardized techniques.^1^

**Table 1:** Agreement and kappa analysis of acceptability and components of pre- and post-BD spirometry in the EPOC.AR study by reviewing team and total.

	Criteria	Nº agreements	% agreement	N° agreements by chance	% agreements by chance	Kappa	95% CI	Qualification
RvTa (n = 4,124)	Acceptability	3148	76.3	2131.7	51.7	0.51	0.48 - 0.54	Moderate
	Quantity	2813	68.2	2032.6	49.3	0.49*	0.35 - 0.4	Moderate
	Defect	2638	64.0	1957.2	47.5	0.31	0.29 - 0.33	Fair
	Repeatability	3287	79.7	2696.5	65.4	0.44	0.38 - 0.45	Moderate
RvTb (n = 3,840)	Acceptability	3214	83.7	2163.4	56.3	0.63	0.6 - 0.65	Substantial
	Quantity	3031	78.9	2101.5	54.7	0.65*	0.51 - 0.56	Substantial
	Defect	2960	77.1	2178.5	56.7	0.47	0.44 - 0.5	Moderate
	Repeatability	3314	86.3	2542	66.2	0.59	0.56 - 0.63	Substantial
TOTAL (n = 7,964)	Acceptability	6362	79.9	4295.4	53.9	0.56	0.54 - 0.58	Moderate
	Quantity	5844	73.4	4152.1	52.1	0.56*	0.43 - 0.46	Moderate
	Defect	5598	70.3	4150.7	52.1	0.38	0.36 - 0.4	Fair
	Repeatability	6601	82.9	5241.4	65.8	0.5	0.48 - 0.52	Moderate

RvTa and RvTb, reviewing teams a and b; CI, confidence interval. Quantity = number of acceptable maneuvers. *Weighted kappa.

Men and women aged ≥40 years were recruited from the six selected cities. The sample was selected through multistage probability cluster sampling based on cartographic area units within each city. One individual per household was invited to participate, and all volunteers provided written informed consent. The exclusion criteria were as follows: mental disorders or impaired decision-making capacity; history of thoracic or abdominal surgery within the preceding three months; institutional residence; current COPD exacerbation; active tuberculosis or acute respiratory infection in the previous month; cardiovascular event in the past three months; pregnancy; active or recent pneumothorax; significant hemoptysis of unknown origin; cerebral, thoracic, or abdominal aneurysm (risk of rupture); retinal detachment; and/or eye surgery within the preceding three months.^10^ ^,^ ^11^ Contraindications to spirometry were also taken into account.^12^

Each city submitted weekly spirometry datasets containing all maneuvers performed by each participant. The datasets were reviewed at a coordinating center comprising two reviewer teams (RvTa and RvTb), each assigned to data from one city per study phase. Each team included one senior reviewer (SR)-with 8 and 24 years of experience, respectively-and two junior reviewers (JRs), each with 3-5 years of experience. All reviewers were board-certified pulmonologists trained in high-volume spirometry centers. Each reviewer had full access to each participant’s raw spirometry files, including all measured variables and corresponding flow-volume and volume-time curves for each maneuver. 

Weekly datasets were managed as follows: upon receipt, the SR in each team duplicated the dataset and distributed copies to each JR within their team, along with an electronic evaluation form. The collected variables were: the quality grade automatically assigned by the spirometer software (NDD EasyWare v.2.25.0.0; Zurich, Switzerland), ranging from A to F,^13^ the number of acceptable maneuvers (1, 2, or ≥3), the cause of non-acceptability (1 = no defects; 2 = insufficient expiratory time; 3 = excessive back-extrapolated volume [BEV]; 4 = cough during the first second of expiration; 5 = weak effort; 6 = glottic closure; 7 = other defects), and repeatability (defined as <150 mL difference in FEV_1_ and FVC). Spirometries were classified as acceptable when at least three acceptable maneuvers fulfilled the repeatability criteria.^1^

**Figure 1:**
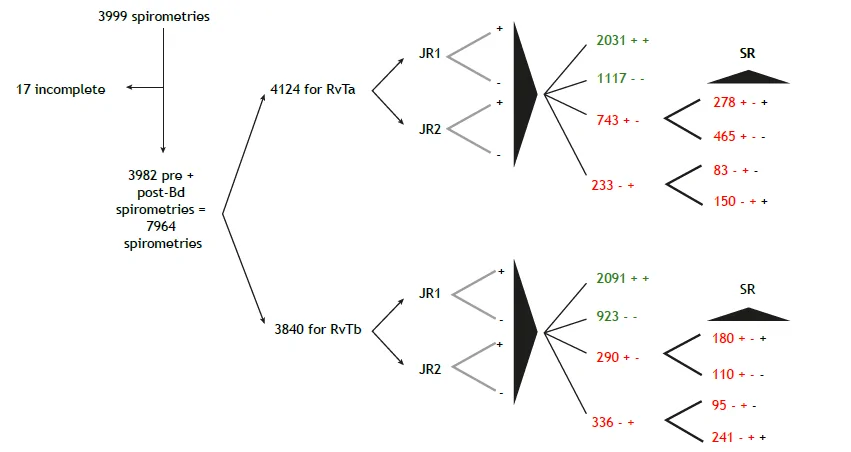
Flow of spirometry data for review in the EPOC.AR study. RvT, reviewing team; JR, junior reviewer; SR, senior reviewer; +, accepted spirometries; -, rejected spirometries. The combination of + and - symbols reflects the distribution of the reviewers’ decisions (e.g., + and - indicates that JR1 accepted and JR2 rejected the preceding number of tests). Green items indicate agreement, while red items indicate disagreement. Black symbols on the right margin indicate SR adjudication of disagreements.

The JRs were blinded to each other’s reviews. After completing their evaluations, each JR submitted their annotated dataset to the SR. Both datasets were then merged into a single electronic form, and a computer algorithm determined agreement or disagreement between the two JRs for each spirometry. Discrepant cases were adjudicated by the SR, who was blinded to the JRs’ individual decisions. Consequently, the acceptability or rejection of each pre- and post-bronchodilator (BD) spirometry was established by agreement among two of the three reviewers (Figure 1). 

The study protocol and informed consent forms were initially reviewed and approved by the Provincial Bioethics Committee of Rosario City, Argentina (Protocol No. 344; approval date: July 23, 2014). Subsequently, the ethical, regulatory, educational, and research aspects of the study were reviewed and approved by regional and local ethics committees from each participating urban area. All participants provided written informed consent.

For the statistical analysis, pre- and post-BD maneuvers were analyzed as separate spirometries and treated individually. Agreement between JRs within each RvT was assessed using Cohen’s kappa coefficient, interpreted across five levels of concordance: 0-0.20, slight agreement; 0.21-0.40, fair agreement; 0.41-0.60, moderate agreement; 0.61-0.80, substantial agreement; 0.81-1.00, almost perfect agreement).^14^ ^,^ ^15^ Weighted kappa was applied to evaluate agreement regarding the number of acceptable maneuvers. Chi-squared tests were used to compare the frequency of spirometric defects between reviewers, and McNemar’s test was applied to analyze proportions of agreement and disagreement between SRs and JRs.

## RESULTS

From the initial pool, 3,982 participants had complete pre- and post-BD spirometry results evaluated by at least two reviewers. Volunteer demographics are presented in Tables 1 and S1. Because pre- and post-BD spirometries were analyzed separately, the total number of tests was 7,964: 4,124 reviewed by RvTa and 3,840 by RvTb. 

Agreement on spirometric acceptability and its components (number of acceptable maneuvers, predominant defects, and repeatability) is summarized in Table 1. Overall agreement across both reviewer teams reached 79.9%. The number of acceptable maneuvers matched in 73.4% of cases, the predominant defect in 70.3%, and repeatability between the two best acceptable blows in 82.9% (bottom rows in Table 1). Separate results for the pre- and post-BD tests are provided in the Supplementary Material (Tables S3 and S4), showing similar patterns across both phases and teams. Concordance for the predominant defect type between both pairs of JRs is shown in Table S5.

**Figure 2:**
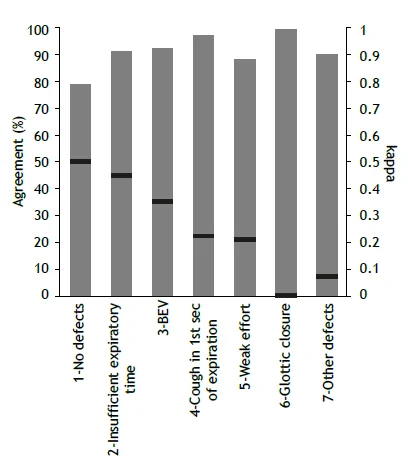
Agreement and kappa analysis of acceptability criteria in pre- and post-BD spirometries: total (A) and by reviewing team (B), in the EPOC.AR study. RvTa and RvTb, reviewer teams a and b; BEV, excessive back-extrapolated volume.

Within each team, the JRs most frequently agreed on the predominant defect, although much of this concordance appeared to occur by chance (Figure 2, left panel). Accordingly, Cohen’s kappa values were relatively low (≤0.5 when “No defect” was considered and ≤0.61 when it was excluded) (Figure S1, left panel). These findings were consistent between both RvTs (Figure 2, right panel; Figure S1, right panel). Among the specific defect categories, the highest agreement was observed for “Insufficient expiratory time” (k=0.61) and “BEV” (k=0.52), whereas the lowest agreement occurred for “Weak effort” (k=0.19) and “Cough during first second of expiration” (k=0.36). Notably, no agreement was observed for the “Glottic closure” category (k=0).

**Figure 3:**
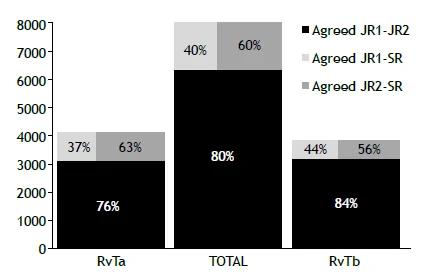
Proportion of agreement and disagreement between junior reviewers, and the proportion of decisions favoring each one, for pre- and post-BD spirometries in the EPOC.AR study. RvTa and RvTb, reviewer teams a and b; JR, junior reviewer; SR, senior reviewer. Agreement between JR1-SR and JR2-SR was significant (McNemar’s p<0.001) for both RvTa and RvTb.

Agreement on overall spirometric acceptability between JR1 and JR2 was 76% in RvTa (3,148/4,124 tests) and 84% in RvTb (3,214/3,840 tests). In cases of disagreement, the SR favored JR1’s decision in 37% (361/976) and JR2’s decision in 63% (615/976) of cases in RvTa, while in RvTb, the SR favored JR1’s decision in 44% (275/626) and JR2’s in 56% (351/626) (Figure 3; McNemar’s p<0.001). Separate data for pre- and post-BD spirometries are presented in Supplementary Figure S2.

**Figure 4:**
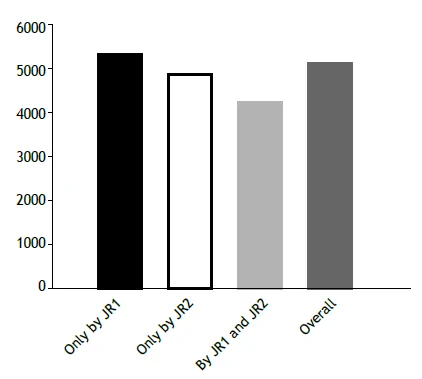
Percentage of spirometric acceptability according to single and combined reviewers in the EPOC.AR study. Overall review corresponds to approval by at least two out of three reviewers. JR, junior reviewer.

Overall, 5,355 of the 7,964 tests (67.3%) were accepted by both JR1 reviewers and 4,891 (61.3%) by both JR2 reviewers. When only tests accepted by both JR1 and JR2 were considered, the number of spirometries eligible for final analysis dropped to 4,322 (54.3%). Subsequent SR adjudication increased this number to 5,171 (64.9%) (Figure 4).

## DISCUSSION

Spirometric acceptability is defined by the performance of at least three acceptable maneuvers, the best two of which must be repeatable. These criteria have gradually evolved based on evidence and expert consensus to ensure reliable results.^1^ ^,^ ^2^ Although they have been in use for the past twenty years, we hypothesized that it would be relevant to test their reliability when assessed by different reviewers. 

While each spirometry included a software-assigned quality grade, the reviewers were instructed to disregard it and assess each test manually. Disagreement between reviewers was observed not only in overall spirometric acceptability but also in its individual components. 

Apparently, an acceptable spirometry was relatively easy for the reviewers to identify (Figure 2; Table S5). Nevertheless, the predominant technical defect was interpreted differently by the JRs in 36% and 23% of cases in RvTa and RvTb, respectively (Table 1). This finding is not unexpected, as there were seven possible defect categories, and many of the technical defects described in the guidelines involve subjective judgement-such as “Effort that is not maximal throughout” or “Coughing in the first second or any other cough that, in the technician’s judgment, interferes with the measurement of accurate results.”^1^ However, even more objective criteria-such as “BEV” or “Insufficient expiratory time” (defined in the statement as “no change in volume (0.025 L) for ≥1 second, and the subject has tried to exhale […] for ≥6 seconds in subjects aged >10 years”)-also showed disagreement (Table S5). 

The criteria showing the highest agreement were “Insufficient expiratory time” and “BEV”, probably because they are numerical measures. In contrast, “Cough during the first second of expiration” and “Weak effort”, which rely mainly on visual inspection and are therefore more prone to subjectivity, exhibited lower agreement. “Glottic closure” was almost absent among nearly 8,000 tests analyzed. A plausible explanation is that glottic closure occurs more frequently in younger individuals, whereas all participants in this study were 40 years or older. Furthermore, the fact that this study was conducted in referral centers with trained personnel likely helped minimize the occurrence of this rare defect. 

Interestingly, “BEV” and “Weak effort” were interpreted differently between the two RvTs, probably because these criteria are interdependent, but also due to the poor definition of what constitutes a “Weak effort”. Reviewers were asked to tag the predominant defect found in each test. Since the number of acceptable maneuvers was judged differently by the JRs, the predominant defect could vary depending on each reviewer’s assessment. Although the software-assigned quality grade was embedded in each spirometry, reviewers were instructed to ignore it and assess each test manually.

Another notable finding was the substantial inter-reviewer disagreement regarding the number of acceptable maneuvers (32% and 21%) and repeatability (20% and 14%), especially considering that the latter appears to be largely numerical at first glance. This may be explained by the selection of different maneuvers as the top two in each test (see Table 1). 

In 2018, Velickovski et al.^16^ reported agreement levels in the context of an automated algorithm, with kappa values ranging from 0.19 (52%) to 0.53 (86%) in pairwise comparisons among three experts.^16^ Similarly, Walag et al.^17^ reported kappa values of 0.70 and 0.71 for FEV_1_ and FVC, respectively.^17^ However, in both studies, the analysis was limited to the binary classification of maneuvers as acceptable or non-acceptable. In contrast, our study evaluated not only overall spirometric acceptability but also its individual components. Because each session included up to eight maneuvers to choose from, the likelihood of classifying a spirometry as non-acceptable was lower, which in turn increased the agreement on overall acceptability.

The fact that the SR’s decisions favored one JR more than the other-especially in RvTa (Figure 3)-suggests that retraining should be recommended not only for operators, but also for reviewers. Unlike other pulmonary function tests, spirometry involves numerous acceptability criteria, and both operators and reviewers tend to forget some of them over time.^18^ ^,^ ^19^ Accordingly, many spirometry training programs recommend recertification every three years.^20^ ^,^ ^21^

One strength of this study is that we were able to test our hypothesis in two distinct groups, RvTa and RvTb. The analysis could also be replicated within each group, since pre- and post-BD spirometries were evaluated as separate tests. The study’s internal consistency was demonstrated by the similar agreement proportions observed between pre- and post-BD spirometries for each reviewer team (Supplementary Tables S3 and S4). Another strength is that every maneuver from every test was independently inspected by at least two experienced reviewers, and the final decision on acceptability required the blinded agreement of two out of three reviewers. However, this methodology demands considerable time and human resources. 

Although the sample was limited to individuals over 40 years of age, we believe this restriction did not affect the study’s objectives. Another limitation is that acceptability criteria were assessed globally for each session rather than at the individual maneuver level. Furthermore, only one label could be assigned per test. This simplification of categories was intentionally designed to facilitate the review process given the large number of tests. 

Spirometry acquisition and review procedures followed the 2005 ATS/ERS guidelines.^1^ ^,^ ^12^ The 2019 update was not in use at the time of data collection,^2^ and its implementation has been slow, particularly in clinical settings. Nevertheless, this study underscores the challenges of achieving consensus, even when well-defined and rigorously applied criteria are used by reviewers. 

Our findings show that even among trained personnel, approximately one-quarter to one-fifth of tests were classified differently. This has technical and economic implications, as well as consequences for the design of large-scale studies involving spirometry performance. If tests were reviewed only by the stricter reviewer, many could have been discarded, necessitating a larger population sample and increasing costs. Conversely, reviewing only by the less strict reviewer could lead to the inclusion of marginally acceptable tests, thereby widening the confidence interval and reducing overall data quality (Figure 4). Either scenario could influence sample size calculations and affect the final outcomes. 

In clinical practice, variability among spirometry reviewers may compromise report reliability, as the selection of acceptable maneuvers directly influences the final values of key parameters such as FVC and FEV_1_. Consequently, the classification of spirometric patterns may change, particularly when results are close to the lower limit of normality. Such variability can affect subsequent clinical decisions, including referrals for further or more complex investigations (e.g., lung volume measurements or imaging), ultimately increasing healthcare costs. Moreover, these implications extend to contexts in which spirometric results are determinative, such as occupational health claims, preoperative risk assessment, and eligibility for transplantation waiting lists.

These findings also have implications for the development of automated algorithms. Training artificial intelligence models requires clear and unambiguous input data. Some authors have addressed this challenge by conducting preliminary consensus meetings among experts to standardize quality assessment criteria and jointly review complex cases.^21^

Reviewer variability significantly affects spirometric acceptability, with numerical criteria achieving higher agreement than subjective assessments. Such discrepancies influence not only the quality of large-scale studies but also data integrity and the development of automated algorithms. Greater standardization of spirometric acceptability criteria may therefore be warranted. Future studies using updated standards could yield different results. While automated tools are still under development, cross-reviewing by multiple evaluators remains advisable to maximize data reliability.
